# Supplementary figures and images for: Minimal effects of ultraviolet light supplementation on egg production, egg and bone quality, and health during early lay of laying hens
Source: PeerJ. 2023 Mar 15;11:e14997. doi: 10.7717/peerj.14997 (PMC10024484; doi:10.7717/peerj.14997)

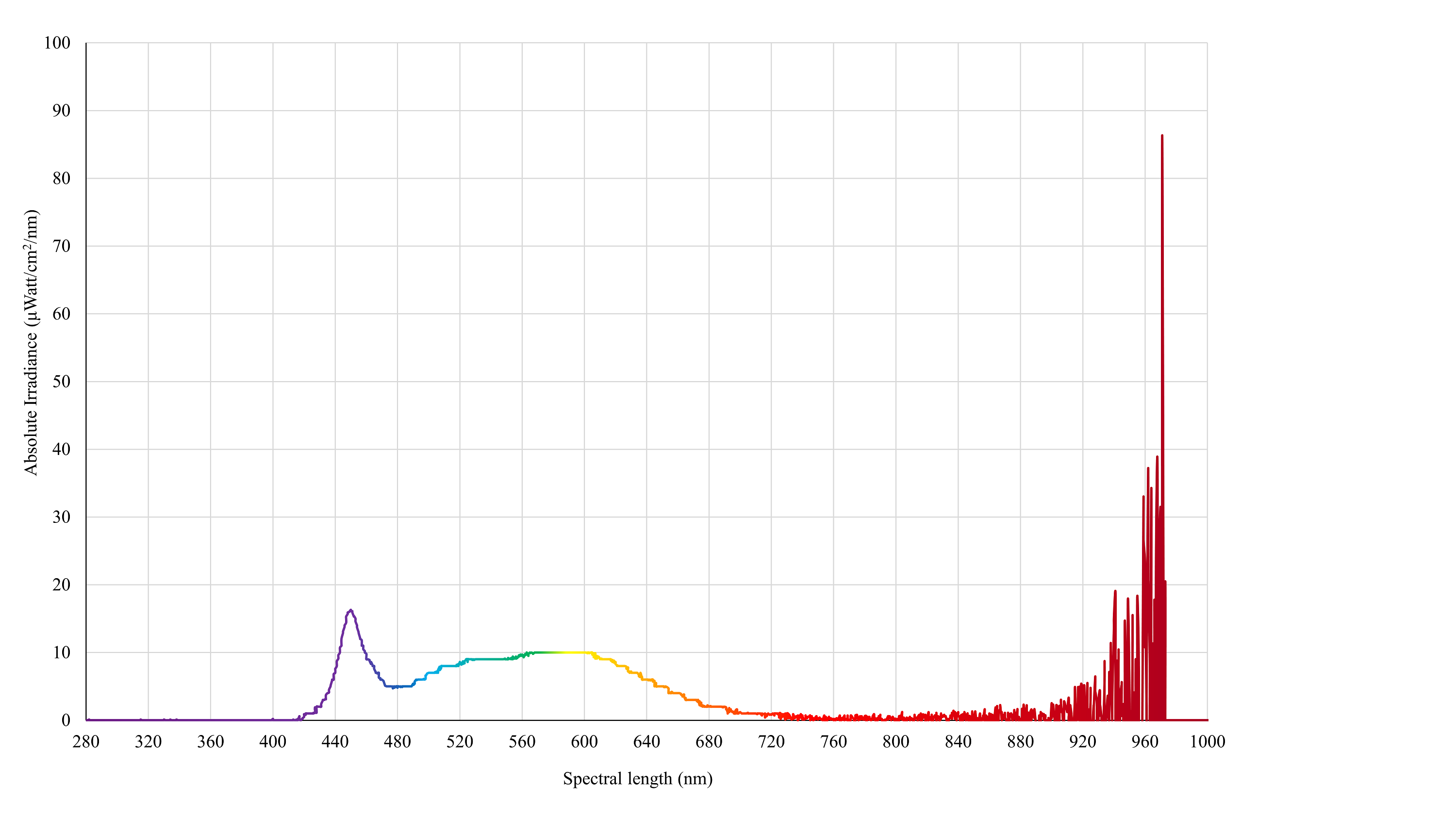

Supplement: Figure S1 — Spectral irradiance of a poultry-specific LED white bulb (IP65 Dimmable LED Bulb, B-E27: 10W, 5K) as measured by an Ocean Insight Flame-S-XR1 Spectroradiometer at 15 cm from source (a closer distance was measured for the control bulb for visual appearance of the readings). [file peerj-11-14997-s001.png]

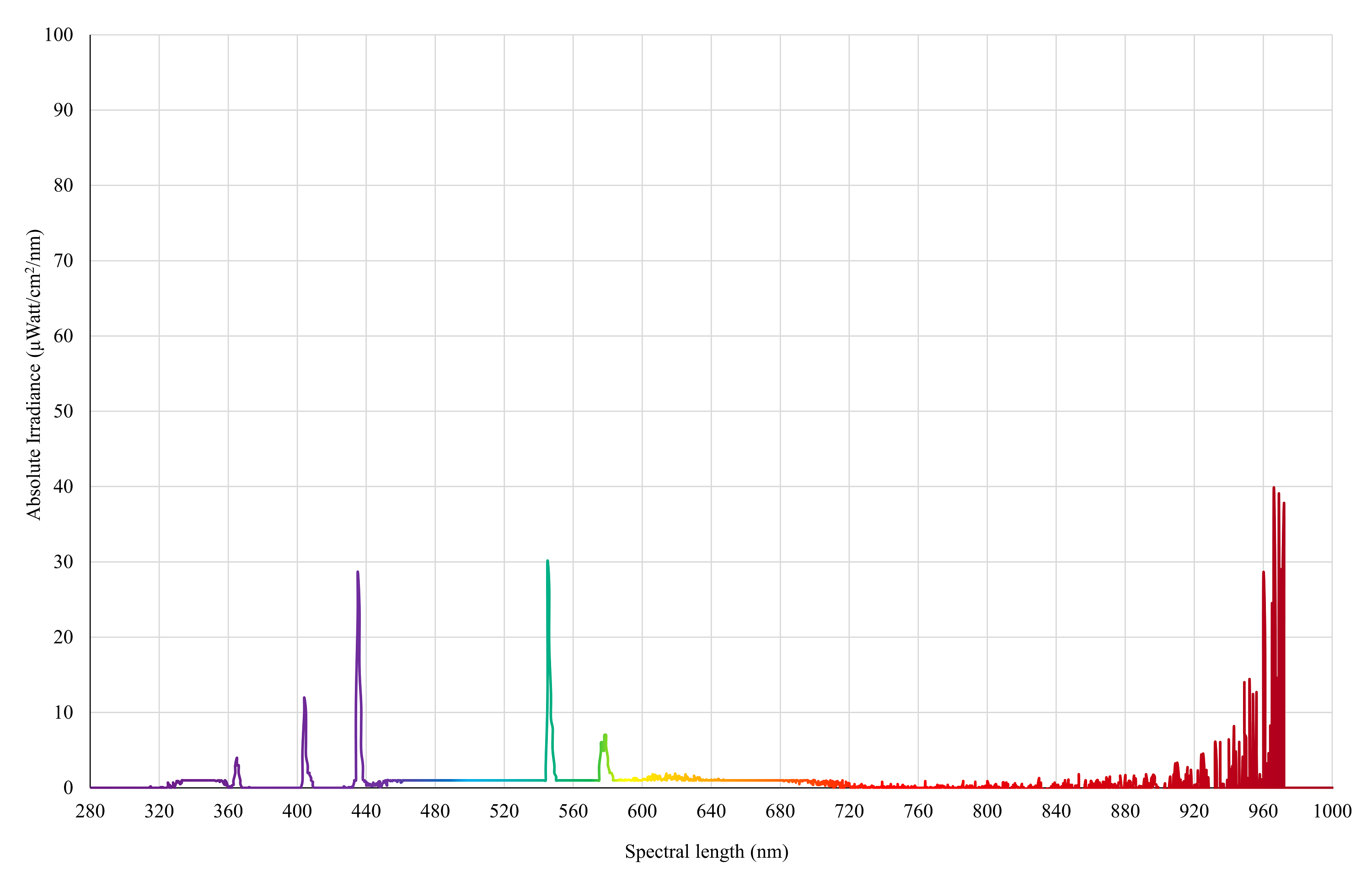

Supplement: Figure S2 — Spectral irradiance of 3 × PureSun Compact Bird Lamps (E27-20W, Arcadia, Germany) with 3-mm glass placed under the bulbs to block UVB spectrum as measured by an Ocean Insight Flame-S-XR1 Spectroradiometer at chicken eye height (30 cm from the floor). [file peerj-11-14997-s002.png]

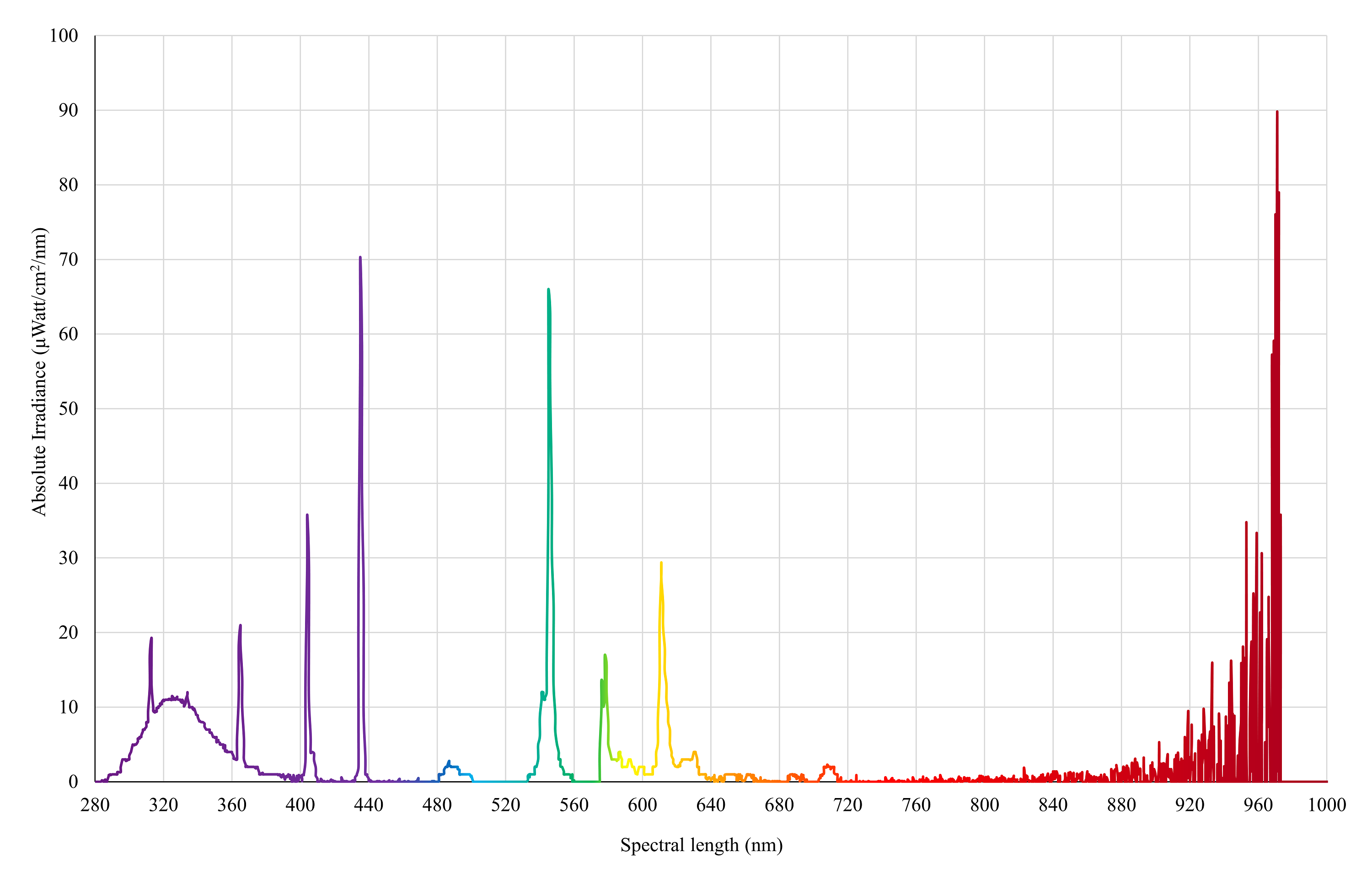

Supplement: Figure S3 — Spectral irradiance of 3 × Exo-Terra® Reptile UVB200 lights (25W, PT2341, 25W, PT2341, Rolf C. Hagen, Montreal, QC, Canada) as measured by an Ocean Insight Flame-S-XR1 Spectroradiometer at chicken eye height (30 cm from the floor). [file peerj-11-14997-s003.png]

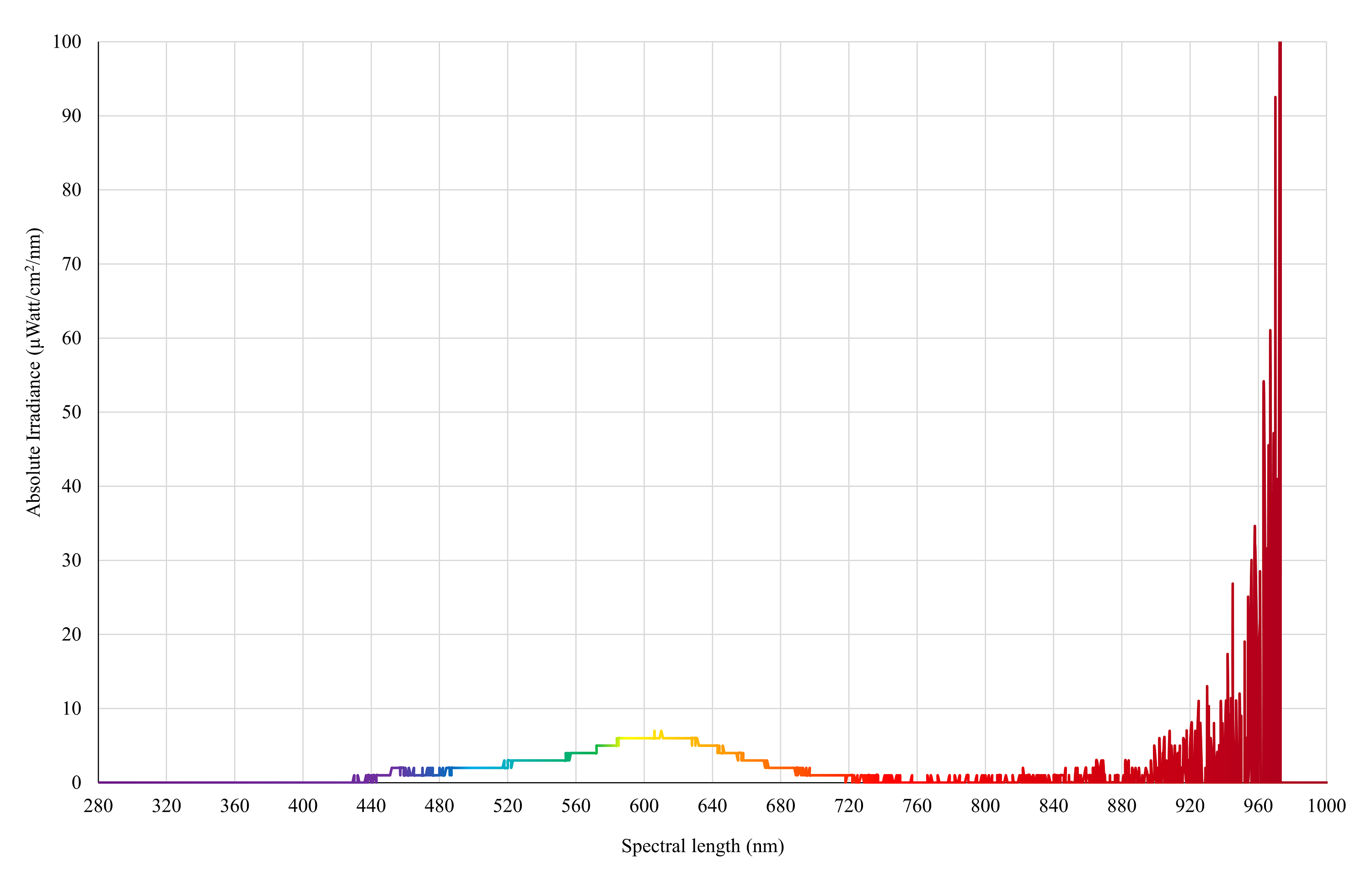

Supplement: Figure S4 — Spectral irradiance of 3 × LED Filament Warm-white lights (Edison Screw LED GLS Filament Globe, E27-4W, Mirabella, Victoria, Australia) as measured by an Ocean Insight Flame-S-XR1 Spectroradiometer at chicken eye height (30 cm from the floor). [file peerj-11-14997-s004.png]
